# Comparison of Long-term Survival Benefits in Trials of Immune Checkpoint Inhibitor vs Non–Immune Checkpoint Inhibitor Anticancer Agents Using ASCO Value Framework and ESMO Magnitude of Clinical Benefit Scale

**DOI:** 10.1001/jamanetworkopen.2019.6803

**Published:** 2019-07-10

**Authors:** Louis Everest, Monica Shah, Kelvin K. W. Chan

**Affiliations:** 1 Odette Cancer Centre, Sunnybrook Health Sciences Centre, Toronto, Ontario, Canada; 2 University of Toronto, Toronto, Ontario, Canada; 3 Canadian Centre for Applied Research in Cancer Control, Toronto, Ontario, Canada; 4 Cancer Care Ontario, Toronto, Ontario, Canada

## Abstract

**Importance:**

Recently, anticancer agents have generated excitement owing to their capacity to preserve long-term durable survival in some patients who are represented by a tail of the survival curve. However, because traditional measures of clinical benefit may not accurately capture durable survival, amendments to various valuation frameworks have been proposed to capture this benefit.

**Objectives:**

To determine how frequently immune checkpoint inhibitor (ICI) anticancer agents vs non-ICI anticancer agents displayed trends of long-term durable survival, as defined by the American Society of Clinical Oncology Value Framework version 2 (ASCO-VF v2) and European Society of Medical Oncology Magnitude of Clinical Benefit Scale version 1.1 (ESMO-MCBS v1.1), as well as to further analyze the degree of agreement between ASCO and ESMO frameworks.

**Design, Setting, and Participants:**

In this cohort study, anticancer agents from phase 2 or 3 randomized clinical trials (RCTs) cited for clinical efficacy evidence in drug approval by the US Food and Drug Administration between January 2011 and March 2018 were identified. Data required for the ASCO-VF v2 tail-of-the-curve bonus and the ESMO-MCBS v1.1 immunotherapy-triggered long-term plateau adjustments were extracted from relevant RCTs. Frequency and difference in proportions were calculated to determine how often survival benefits were awarded to anticancer agents overall and to ICI and non-ICI anticancer agents individually.

**Main Outcomes and Measures:**

American Society of Clinical Oncology Value Framework v2 tail-of-the-curve bonuses and ESMO-MCBS v1.1 immunotherapy-triggered long-term plateau adjustments.

**Results:**

In total, 247 RCTs were identified, and 100 RCTs involving 57 164 patients were included, with 14 examining ICI agents (1 ipilimumab, 5 pembrolizumab, 5 nivolumab, 2 atezolizumab, and 1 durvalumab) and 86 examining non-ICI agents (74 targeted therapy, 8 chemotherapy, 3 hormone therapy, and 1 radiopharmaceutical). Randomized clinical trials were awarded ASCO-VF v2 tail-of-the-curve bonuses more often than ESMO-MCBS v1.1 immunotherapy-triggered long-term plateau adjustments (ASCO-VF v2, 45.0% [8 of 14 ICI RCTs and 37 of 86 non-ICI RCTs] vs ESMO-MCBS v1.1, 2.6% [1 of 12 ICI RCTs and 1 of 66 non-ICI RCTs). Randomized clinical trials for ICIs were not more likely to receive an ASCO-VF v2 bonus or ESMO-MCBS v1.1 adjustment than non-ICI RCTs (ASCO-VF: risk difference, 0.14; 95% CI, −0.14 to 0.42; *P* = .32; ESMO-MCBS: risk difference, 0.07; 95% CI, −0.09 to 0.23; *P* = .40). Poor agreement was found between the framework algorithms in identifying long-term survival benefits from RCTs (κ = 0.01; 95% CI, −0.23 to 0.22; *P* = .50).

**Conclusions and Relevance:**

The ASCO-VF v2 and ESMO-MCBS v1.1 may require additional refinement to accurately capture the benefit of durable long-term survival, or ICI agents may not preserve long-term survival as conventionally thought.

## Key Points

**Question:**

How frequently do immune checkpoint inhibitor (ICI) agents vs non-ICI agents qualify for American Society of Clinical Oncology Value Framework version 2 (ASCO-VF v2) tail-of-the-curve bonuses or European Society of Medical Oncology Magnitude of Clinical Benefit Scale version 1.1 (ESMO-MCBS v1.1) immunotherapy-triggered long-term plateau adjustments?

**Findings:**

In this cohort study of 100 randomized clinical trials comparing ICI with non-ICI agents, ASCO-VF v2 tail-of-the-curve bonuses and ESMO-MCBS v1.1 immunotherapy-triggered long-term plateau adjustments were not awarded to ICI agents more often than non-ICI agents.

**Meaning:**

The ASCO-VF v2 and ESMO-MCBS v1.1 may be flawed in accurately capturing long-term survival.

## Teaser

This study of 100 randomized clinical trials determines how frequently immune checkpoint inhibitor (ICI) and non-ICI anticancer agents displayed trends of long-term durable survival as defined by the American Society of Clinical Oncology (ASCO) Value Framework and the European Society of Medical Oncology (ESMO) Magnitude of Clinical Benefit Scale.

## Introduction

Conventional non–immune checkpoint inhibitor (ICI) anticancer agents typically improve patients’ overall survival (OS) and progression-free survival (PFS).^1^ However, long-term survival benefits are limited by acquired biological resistance.^2^ In contrast to non-ICI agents, ICI agents have the potential to show long-term survival, represented by a plateau at the tail of the survival curve in small patient populations for cancer types including melanoma.^3^ While ICI agents are often preferred as treatment options,^4^ they are often costlier than non-ICI agents.^5^ As such, there are concerns regarding the relationship between price and clinical benefit. Saluja et al^6^ have demonstrated that while the costs of novel oncology drugs have risen during the last decade, the clinical benefits of these medications have not experienced a proportional increase. Efforts to evaluate the association of efficacy with the cost of novel therapies have led to the development of various value frameworks.^7^

To objectively quantify therapy value and clinical benefit, the American Society of Clinical Oncology Value Framework (ASCO-VF)^8,9^ and the European Society for Medical Oncology Magnitude of Clinical Benefit Scale (ESMO-MCBS)^10,11^ have been proposed as valuation frameworks that analyze survival, toxicity, and quality-of-life measures. However, literature suggests that conventional measures of treatment effect, including median survival and hazard ratios, do not accurately capture the tail of the survival curve in randomized clinical trials (RCTs) with long-term survival populations.^12,13^ To more accurately measure long-term survival benefits, ASCO-VF and ESMO-MCBS amendments were made to incorporate bonuses and adjustments that capture the tail of the survival curve; ASCO-VF version 2 now incorporates a tail-of-the-curve (TOC) bonus awarded for long-term OS or PFS in the experimental arm,^9^ and ESMO-MCBS version 1.1 now incorporates various immunotherapy-triggered adjustments, including long-term plateau (LTP) adjustments awarded for plateauing in OS and PFS survival curves.^11^

Furthermore, 3 additional frameworks have been proposed to assess the value of novel anticancer agents: the National Comprehensive Cancer Network framework,^14^ the Institute for Clinical and Economic Review framework,^15^ and the DrugAbacus framework developed by the Memorial Sloan Kettering Cancer Center.^16^ While these frameworks use various methods to assess value, none directly evaluate long-term survival.^7,17^

Although ASCO-VF and ESMO-MCBS aim to capture long-term survival benefits, their respective criteria and award values vary and may be perceived as arbitrary. For example, to credit plateauing in PFS, ASCO-VF v2 awards 16 points as a TOC bonus,^9^ while ESMO-MCBS v1.1 awards an upgrade of 1 level as 1 of 2 immunotherapy-triggered LTP adjustments.^11^ Further, ASCO-VF v2 provides numerical final scores and describes substantial benefit^18^ as a score more than 45.^9^ In contrast, ESMO-MCBS v1.1 provides ranked grades of 1 to 5 for the advanced disease setting and assigns grades of C, B, or A for the curative setting, describing meaningful clinical benefit^10^ as a grade of 4, 5, B, or A.^11^

Owing to framework variability and the importance of ensuring ASCO-VF and ESMO-MCBS amendments are valid in measuring long-term survival benefits, Cherny et al^18^ conducted a comparative assessment of ASCO-VF v2 and ESMO-MCBS v1.1 to evaluate their concordance in measuring clinical benefit. In this assessment, a Spearman rank correlation coefficient of 0.68 was calculated for paired scores in 102 RCTs.^18^ Cherny et al^18^ suggested that a major factor contributing to the discordance found between 37 of 102 RCTs may be the crediting of the tail of the survival curve, accounting for 8 of 37 discordant studies.^18^

Although it has been shown that ASCO-VF v2 and ESMO-MCBS v1.1 differ in their scoring outcomes when awarding a TOC bonus or LTP adjustment^18^ owing to the absence of a criterion-standard definition of the tail of the survival curve, it is unclear which framework is better developed or defined to more accurately capture long-term survival benefits. Challenges in developing a criterion-standard definition of tail of the survival curve include the variable effects of ICIs on different cancers. For example, compared with hormone receptor–positive breast cancer, melanoma displays increased sensitivity to ICI anticancer agents.^19^ As such, the extent to which certain ICIs and non-ICIs are identified as having long-term survival benefit by ASCO and ESMO frameworks may depend on the cancer type treated.

As ICI agents are believed to have the potential for long-term survival in contrast to non-ICI agents, it is important to assess whether ICI RCTs are awarded long-term survival benefits more often than non-ICI RCTs by ASCO and ESMO valuation frameworks. Thus, the objectives of this study were not only to examine how frequently RCTs qualified for an ASCO-VF v2 TOC bonus and ESMO-MCBS v1.1 immunotherapy-triggered LTP adjustments but also to examine how often these bonuses and adjustments were awarded specifically to ICI vs non-ICI RCTs.

## Methods

The US Food and Drug Administration (FDA) Hematology/Oncology Approvals and Safety Notifications page was reviewed to identify all RCTs cited for clinical efficacy evidence in oncology drug approvals between January 2011 and March 2018.^20^ Notifications regarding marketing approvals, changes to drug packaging, optical imaging tests, treatment for adverse effects, biosimilars, nononcology conditions, dosage changes, and announcements were excluded from analysis. Approvals citing phase 2 (comparative) or phase 3 RCTs reporting primary (or coprimary) end points of OS and/or PFS that displayed statistically significant improvement in the primary or secondary end point were eligible for inclusion. All relevant RCTs were retrieved from the PubMed database. Study characteristics are summarized in Table 1. Upon retrieval, RCTs were evaluated for qualification of long-term survival benefits using ASCO and ESMO framework criteria. Award criteria are summarized in Table 2. The present study involved publicly available data. Per the Sunnybrook Research Institute Research Ethics Board Standard of Practice, this study did not require an ethics committee review.

**Table 1.** Characteristics of 100 Included Studies

Characteristic	No. (%)
Year	
2008	1 (1.0)
2009	1 (1.0)
2010	4 (4.0)
2011	2 (2.0)
2012	18 (18.0)
2013	8 (8.0)
2014	13 (13.0)
2015	17 (17.0)
2016	21 (21.0)
2017	14 (14.0)
2018	1 (1.0)
Primary end point	
Overall survival	33 (33.0)
Progression-free survival	58 (58.0)
Overall survival and progression-free survival	9 (9.0)
Disease site	
Genitourinary	15 (15.0)
Gastrointestinal	14 (14.0)
Breast	10 (10.0)
Hematologic	21 (21.0)
Lung	17 (17.0)
Thyroid	4 (4.0)
Skin	11 (11.0)
Liposarcoma	4 (4.0)
Squamous-cell	2 (2.0)
Neuroendocrine	2 (2.0)
Therapy type	
Chemotherapy	8 (8.0)
Targeted agent	74 (74.0)
Immune checkpoint inhibitor	14 (14.0)
Hormone therapy	3 (3.0)
Radiopharmaceutical	1 (1.0)

**Table 2.** Characteristics of Value Framework Algorithms

Method	ASCO-VF v2 Tail-of-the-Curve Bonus		ESMO-MCBS v1.1 Form 2b Immunotherapy-Triggered Long-term Plateau Adjustment	ESMO-MCBS v1.1 Form 2a Immunotherapy-Triggered Long-term Plateau Adjustment
End point evaluated	OS	PFS	OS	PFS
Long-term survival definition	Numeric definition of tail of the curve		Qualitative definition of plateau	
Time(s) evaluated	OS at time 2 × median OS of standard regimen	PFS at time 2 × median PFS of standard regimen	5 y: Median OS of control ≤12 mo	1 y: Median PFS of control ≤6 mo
			7 y: Median OS of control >12 mo	2 y: Median PFS of control >6 mo
Criterion 1 of 2	≥50% Improvement in proportion of patients alive with the test regimen	≥50% Improvement in proportion of patients progression free with the test regimen	Long-term plateau of the OS curve	Long-term plateau of the PFS curve
Criterion 2 of 2	Patients surviving ≥20% with standard regimen	Patients progression free ≥20% with standard regimen	OS advantage in the test regimen	10% or greater improvement in PFS
Bonus awarded	20 bonus points	16 bonus points	Scored with form 1 (receipt of additional letter grade representing curative potential)	Upgrade 1 clinical benefit grade level

Abbreviations: ASCO-VF v2, American Society of Clinical Oncology Valuation Framework, version 2; ESMO-MCBS v1.1, European Society of Medical Oncology Magnitude of Clinical Benefit Scale, version 1.1; OS, overall survival; PFS, progression-free survival.

As stated in ASCO-VF v2,^9^ TOC bonuses of 20 points for OS and 16 points for PFS are awarded when the following 4 criteria are met: (1) the RCT reports OS or PFS data, (2) the RCT reports OS or PFS data at twice the standard regimen median OS or PFS, (3) there is 50% or greater improvement in patients alive with the test regimen at time point 2, and (4) 20% or more patients survive with the standard regimen at time point 2. Thus, to determine whether TOC bonus criteria were met, the following data were extracted from RCT survival curves: median OS or PFS and OS or PFS at time 2 × median OS or PFS.

While ASCO-VF v2 evaluates eligibility for the TOC bonus primarily in OS, their algorithm details that if OS is not reported, an RCT does not qualify for an OS-evaluated bonus. If OS determination is obscured by trial design (eg, if crossover was permitted in the comparator arm), then PFS is evaluated.^9^ Further, only 1 TOC bonus may be awarded per study.^9^ When both OS and PFS criteria are met, only an OS-evaluated bonus of 20 points is awarded.^9^

As stated in ESMO-MCBS v1.1,^11^ 2 immunotherapy-triggered LTP adjustments are now awarded based on long-term OS and PFS improvement. While the motivation to include these amendments was triggered by identified shortcomings in the evaluation of ICI RCTs using the original ESMO-MCBS framework, they are also applicable to the evaluation of non-ICI RCTs.

When an immunotherapy-triggered LTP adjustment is awarded on the basis of PFS data, the final clinical benefit grade is upgraded by 1 point.^11^ This occurs when the following 2 criteria are met: (1) LTP in the PFS curve and (2) an improvement of 10% or greater in PFS at 1 year (median control regimen ≤6 months) or 2 years (median control regimen >6 months). When an OS-based immunotherapy-triggered LTP adjustment is awarded, RCTs are scored with form 1, which potentially grants a curative clinical benefit grade (A, B, or C) in addition to the clinical benefit grade awarded without the adjustment (ie, A/4).^11^ This occurs when the 2 following criteria are met: (1) LTP in the OS curve and (2) advantage of the test regimen observed in the OS curve at 5 years (median control regimen ≤12 months) or 7 years (median control regimen >12 months).^11^ Thus, to determine whether immunotherapy-triggered LTP adjustment criteria were met, the following data were extracted from RCT survival curves: lower 95% CIs of the OS or PFS hazard ratio, median OS or PFS, PFS at 1 and 2 years, OS duration, and LTP of the OS or PFS curve.

When RCTs had been previously field-tested by the ESMO-MCBS v1.1 developers, the LTP adjustment evaluation in the field-testing of ESMO-MCBS v1.1 was used. However, when not previously field-tested, LTP was assessed qualitatively by 2 independent researchers. Cohen κ correlation statistic of agreement between the researchers was calculated.

Randomized clinical trials that did not meet ESMO-MCBS v1.1 scoring criteria, including RCTs without statistically significant improvements of primary end points or RCTs examining hematological anticancer agents,^10,11^ were not assessed for immunotherapy-triggered LTP adjustments. Conversely, the ASCO-VF v2 scoring criteria permits assessment with hematological anticancer agents and does not require statistically significant improvements for assessment.^8,9^

The ESMO-MCBS v1.1 immunotherapy-triggered LTP adjustments are mutually exclusive in evaluation of OS and PFS end points (ie, an RCT cannot be evaluated for both OS and PFS end points). Conversely, the ASCO-VF v2 may assess both OS and PFS end points in a single RCT.

### Statistical Analysis

Primary analysis examined the frequency of the awarded ASCO-VF v2 TOC bonus and 2 ESMO-MCBS v1.1 immunotherapy-triggered LTP adjustments using framework-specified end points. The framework-specified end point was defined as the end point ASCO-VF v2 and ESMO-MCBS v1.1 used to assess the respective long-term survival bonus or adjustments according to their framework algorithm. Tail-of-the-curve bonuses and immunotherapy-triggered LTP adjustments awarded using the framework-specified end points were also stratified into OS and PFS subgroups in sensitivity analyses.

We also examined how frequently ICI and non-ICI RCTs qualified for the ASCO-VF TOC bonus and ESMO-MCBS immunotherapy-triggered LTP adjustments. Differences in frequency were tested via the risk differences between ICI and non-ICI RCTs qualifying for these bonuses and adjustments as well as a sensitivity analysis in OS and PFS subgroups (eTable 1 in the Supplement). The level of statistical significance used for risk differences was .05, and tests were 2-tailed.

Agreement between ASCO-VF v2 and ESMO-MCBS v1.1 in awarding long-term survival benefits in individual RCTs was calculated via Cohen κ statistics, where 0 indicates agreement equivalent to chance and 1 indicates perfect agreement.^21^ The Cohen κ statistic was calculated in RCTs using the framework-specified end point and in OS and PFS subgroups. The Cohen κ statistic was used in the present analysis because TOC bonuses and immunotherapy-triggered LTP adjustments are binary. Additionally, agreement was calculated via McNemar χ^2^ test (eTable 2 in the Supplement). The level of statistical significance used for Cohen κ statistic and McNemar χ^2^ test was .05, and tests were 2-tailed. A sensitivity analysis of how frequently ICI and non-ICI RCTs qualified for the ASCO-VF TOC bonus and ESMO-MCBS immunotherapy-triggered LTP adjustments across cancer type was conducted. All analysis was conducted using R version 3.5.0 (The R Foundation).

## Results

**Figure 1.**
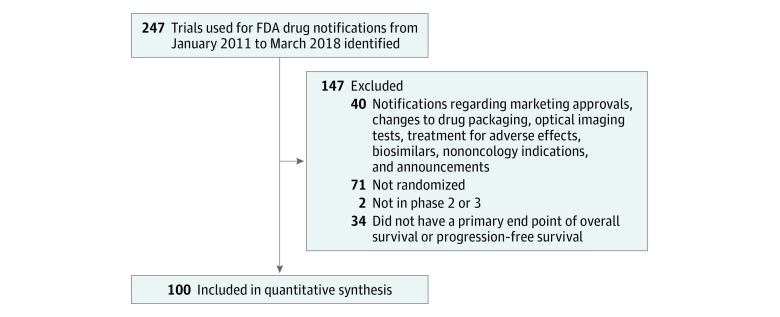
Flow Diagram of Included Studies FDA indicates Food and Drug Administration.

In total, 247 FDA approval indications were identified. Overall, 207 of 247 approval notifications cited trials for FDA approval. Of these, 71 were excluded for lacking randomization and 36 for failing to meet inclusion criteria (Figure 1). A total of 100 RCTs involving 57 164 patients were analyzed, with 14 examining ICI agents (1 ipilimumab, 5 pembrolizumab, 5 nivolumab, 2 atezolizumab, and 1 durvalumab) and 86 examining non-ICI agents (74 targeted therapy, 8 chemotherapy, 3 hormone therapy, and 1 radiopharmaceutical) (Table 1).

**Figure 2.**
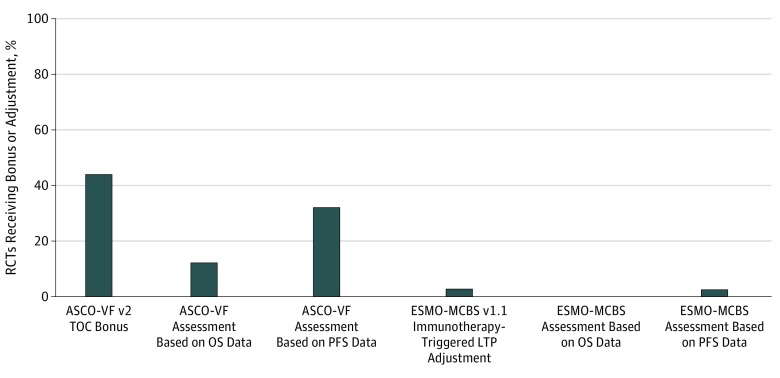
Randomized Clinical Trials (RCTs) That Qualified for Bonuses and Adjustments ASCO-VF v2 indicates American Society of Clinical Oncology Value Framework version 2; ESMO-MCBS v1.1, European Society of Medical Oncology–Magnitude of Clinical Benefit Scale version 1.1; LTP, long-term plateau; OS, overall survival; PFS, progression-free survival; and TOC, tail of the curve.

Eligibility for the ASCO-VF v2 TOC bonus was analyzed in 100 RCTs. This bonus was awarded to 45 of 100 RCTs (45.0%) (Figure 2); 8 of 14 ICI RCTs (57.1%) and 37 of 86 non-ICI RCTs (43.0%) were awarded the TOC bonus (eFigure 1 and eFigure 2 in the Supplement). No statistically significant difference in proportions was found between ICI and non-ICI agents receiving these bonuses using the framework-specified end points (risk difference, 0.14; 95% CI, −0.14 to 0.42; *P* = .32) (eTable 1 in the Supplement).

Of 100 RCTs included in the analysis, 78 were eligible for assessment for an ESMO-MCBS v1.1 immunotherapy-triggered LTP adjustment. This adjustment was awarded to 2 of 78 RCTs (2.6%) (Figure 2). Overall, 1 of 12 ICI RCTs (8.3%) and 1 of 66 non-ICI RCTs (1.5%) were awarded the immunotherapy-triggered LTP adjustment (eFigure 3 and eFigure 4 in the Supplement). No statistically significant difference in proportions was found between ICI and non-ICI agents receiving these adjustments using framework-specified end points (risk difference, 0.07; 95% CI, −0.09 to 0.23; *P* = .40) (eTable 1 and eTable 2 in the Supplement). The ESMO-MCBS v1.1 immunotherapy-triggered LTP adjustments were assessed using data from ESMO-MCBS v1.1 field-testing in 45 RCTs and by investigator review in 33 RCTs.

Of 100 RCTs included in the study, 78 RCTs qualified for evaluation of long-term survival benefits with both ASCO-VF v2 and ESMO-MCBS v1.1. Among these, framework algorithms were in agreement in 40 RCTs (51.3%): 1 agreement (2.5%) for awarding a bonus or adjustment and 39 agreements (97.5%) for withholding a bonus or adjustment. Framework algorithms were in disagreement on the remaining 38 RCTs (48.7%); 1 RCT (2.6%) received an ESMO-MCBS adjustment but not an ASCO-VF bonus, and 37 RCTs (97.4%) received ASCO-VF bonuses but not ESMO-MCBS adjustments.

When comparing the dual-eligible 78 RCTs using the algorithms’ framework-specified end points, Cohen κ statistic was calculated as 0.01 (95% CI, −0.23 to 0.22; *P* = .50) (eTable 3 in the Supplement), which suggested poor agreement between the 2 frameworks in identifying long-term survival benefit from RCTs. These results were consistent with the McNemar χ^2^ test (eTable 3 in the Supplement). Of the 78 RCTs that qualified for evaluation by both frameworks, 12 were ICI RCTs and 66 were non-ICI RCTs. The Cohen κ statistics were −0.17 (95% CI, −0.56 to 0.21; *P* = .79) for the 12 ICI RCTs and 0.04 (95% CI, −0.23 to 0.30; *P* = .39) for the 66 non-ICI RCTs when comparing the respective algorithms’ framework-specified end points (eTable 3 in the Supplement). The low Cohen κ statistic in both the ICI and non-ICI groups also suggested poor agreement between the 2 frameworks in identifying the long-term survival benefit in ICI and non-ICI RCTs.

The sensitivity analysis found that the results of the primary analysis were consistent across cancer types: melanoma and non–small cell lung cancer (the remaining ICI cancers types—urothelial, squamous cell, and renal cell carcinoma—were underpowered for sensitivity analysis) (eTable 4 in the Supplement). Reviewer agreement of ESMO-MCBS immunotherapy-triggered LTP adjustments in both OS and PFS was κ = 0.72 (95% CI, 0.54-0.87; *P* < 0.001) (eTable 5 in the Supplement). The table of all included studies is shown in eTable 6 in the Supplement.

## Discussion

In this study, FDA-approved RCTs of ICI and conventional non-ICI anticancer agents were examined for long-term survival benefits defined by the ASCO-VF v2 and ESMO-MCBS v1.1. It was found that long-term survival benefits were awarded to 45.0% of RCTs using the ASCO-VF framework but to only 2.6% using the ESMO-MCBS framework. Interestingly, ICI RCTs were not more likely to be awarded a long-term survival bonus or adjustment than non-ICI RCTs. This finding, in conjunction with poor agreement between ASCO-VF and ESMO-MCBS frameworks in awarding long-term survival benefits, highlights the challenges and potential arbitrariness of each framework’s ability to capture this benefit and the need for a criterion-standard method to identify long-term survival.

Dissimilar to the present analysis, where an ASCO-VF v2 TOC bonus was awarded to 45.0% of RCTs, Ben-Aharon et al^17^ demonstrated that durable survival and response rates of modern anticancer agents were rarely recognized as significant by the ASCO framework. This difference may result from unclear algorithm instructions, resulting in different calculated scores by separate graders when using the same framework. In an Invited Commentary in *JAMA Oncology*,^22^ 2 authors of the ASCO-VF noted a discrepancy between the survival probabilities extracted by Ben-Aharon et al^17^ and by ASCO-VF investigators from a trial examining ipilimumab plus dacarbazine vs dacarbazine plus placebo.^23^ As stated by the ASCO-VF authors, this discrepancy highlights the need for updated, clear, and easy-to-follow framework instructions.^22^

Further, no association was found between RCTs qualifying for an ASCO-VF v2 TOC bonus and RCTs qualifying for an ESMO-MCBS v1.1 immunotherapy-triggered LTP adjustment. This result supports findings from Cherny et al,^18^ who suggested crediting of the TOC as a major factor contributing to nonconvergence. While a paucity of data exists in the study by Cherny et al^18^ regarding the specific correlation between ASCO-VF v2 and ESMO-MCBS v1.1 based solely on TOC award outcomes, the present analysis showed 38 of 78 RCTs (48.7%) that qualified for evaluation of long-term survival benefits with both frameworks disagreed in award outcomes. However, this high frequency of disagreement may partially result from difficulties in applying the framework algorithms. Cherny et al^18^ stated that low associations observed in previous publications may result from an existing learning curve in the correct application of ASCO-VF and ESMO-MCBS frameworks and from many nuances in the analysis and interpretation of RCT results.

The current study demonstrates that a greater number of RCTs qualified for ASCO-VF v2 TOC bonuses than ESMO-MCBS v1.1 immunotherapy-triggered LTP adjustments, similar to the unilateral discordance found by Cherny et al.^18^ This low rate of assignment of immunotherapy-triggered LTP adjustments by ESMO-MCBS v1.1 may suggest that the ESMO-MCBS framework is insensitive or that no long-term survival improvements have occurred. In addition, bonuses and adjustments were not awarded more often to ICI agents than non-ICI agents using framework-specific end points. This may suggest that the ICI RCTs examined do not display trends of long-term survival more often than the non-ICI RCTs examined, contrary to common belief, or that the ASCO-VF and ESMO-MCBS amendments were not developed or defined accurately, sensitively, or specifically to distinguish long-term survival benefits. In the absence of a universally accepted, criterion-standard method to identify these benefits, clinicians, patients, and decision-makers will continue to struggle with interpreting the magnitude of RCT survival benefits.

Ben-Aharon et al^17^ have suggested a possible refinement to the ASCO-VF v2 TOC bonus may be to lower the required threshold of 20% survival in the control group. As stated by ASCO-VF developers, the purpose of the 20% survival threshold is to prevent imprecision as a function of too few patients alive or progression-free at the milestone selected.^22^ In the present analysis, 3 ICI RCTs and 27 non-ICI RCTs failed to qualify for the ASCO-VF v2 TOC bonus because they did not meet the 20% survival threshold. In the RCTs included in the present analysis, lowering the threshold as recommended by Ben-Aharon et al^17^ would result in both more ICI and non-ICI RCTs qualifying for the ASCO-VF v2 TOC bonus. In contrast to the results of Ben-Aharon et al,^17^ this suggests that the 20% threshold may not be limiting the ASCO-VF’s ability to reward long-term survival benefits in ICI RCTs or that ICI and non-ICI RCTs display the same trends of long-term survival.

Current proposed measures of long-term survival include restricted mean survival time (RMST) difference and milestone survival difference.^24^ Restricted mean survival time is defined as the area under the survival curve.^25^ In contrast to conventional measures of treatment effect, RMST is nonparametric (unlike hazard ratios)^25^ and generates treatment estimates based on the entire survival curve (unlike median survival).^25^ Additionally, survival estimates at specific milestones (eg, at 1, 2, or 5 years) may capture shifts in the survival curve important to patient interests.^26^ Restricted mean survival time and milestone survival may also address concerns regarding imprecision through consideration of statistical significance. While RMST and milestone survival measures are not as widely reported in current RCTs as conventional measures of treatment effect like hazard ratios and median survival,^25^ they can be extracted and calculated from the published Kaplan-Meier curves^27^ and may provide both a practical and objective method of estimation of an observed long-term survival benefit for current and next-generation anticancer agents.^25^ Standard inclusion of RMST and milestone survival in RCTs may allow value frameworks to more accurately capture long-term survival benefits. Additionally, simulation studies based on cure models are warranted to establish a criterion-standard definition of the tail of the survival curve.^28^ Also, in simulation studies, the underlying true model is known and may be used to validate current and proposed metrics on long-term survival.^29^

### Limitations

This study had limitations. Study limitations for consideration include the relatively short follow-up times of RCTs cited in the FDA approval indications. The RCTs analyzed were not necessarily designed to show an effect on long-term survival, and some RCTs lacked mature enough data to appropriately assess long-term survival based on the ASCO-VF v2 and ESMO-MCBS v1.1. Moreover, selective populations participating in RCTs cited in FDA approvals may not be representative of the general population; thus, survival outcomes cannot necessarily be inferred from those observed in the selected group in this study.^30^

## Conclusions

The plateau of a survival curve is indicative of a fundamental shift in cancer care from palliative to curative treatments. Capturing long-term survival benefits with frameworks, including the ASCO-VF and ESMO-MCBS frameworks, is an important aspect of assessing current and future anticancer agents. However, the low agreement observed in this study between the ASCO-VF v2 TOC bonus and the ESMO-MCBS v1.1 immunotherapy-triggered LTP adjustments suggests that these frameworks are flawed in accurately capturing treatment effects. Additionally, ASCO-VF v2 and ESMO-MCBS v1.1 did not award respective bonuses and adjustments to ICI agents more often than non-ICI agents for framework-specified end points, suggesting that, contrary to common belief, ICI agents may not preserve long-term survival in the examined RCTs or that, even with updated frameworks, ASCO-VF v2 and ESMO-MCBS v1.1 are unable to capture this benefit.

## Notes

Supplement. **eFigure 1. **ASCO-VF v2 Tail-of-the-Curve Bonus: PFS Percentage Improvement **eFigure 2.** ASCO-VF v2 Tail-of-the-Curve Bonus: OS Percentage Improvement **eFigure 3.** ESMO-MCBS v1.1 Immunotherapy-Triggered Long-term Plateau Adjustment: PFS Percentage Difference at Time Point Evaluated **eFigure 4.** ESMO-MCBS v1.1 Immunotherapy-Triggered Long-term Plateau Adjustment: OS Study Duration **eTable 1.** Primary Analysis of Risk Differences of Immune Checkpoint Inhibitor vs Non–Immune Checkpoint Inhibitor RCTs Qualifying for ASCO-VF v2 Tail-of-the-Curve Bonus and ESMO-MCBS Immunotherapy-Triggered Long-term Plateau Adjustments **eTable 2.** Sensitivity Analysis of Risk Differences of Immune Checkpoint Inhibitor vs Non–Immune Checkpoint Inhibitor RCTs Qualifying for ASCO-VF v2 Tail-of-the-Curve Bonus and ESMO-MCBS Immunotherapy-Triggered Long-term Plateau Adjustments **eTable 3.** Kappa Correlation Statistic for Agreement Between ASCO-VF v2 and ESMO-MCBS v1.1 Tail-of-the-Curve and Long-term Survival Bonuses **eTable 4.** Risk Differences of Immune Checkpoint Inhibitor vs Non–Immune Checkpoint Inhibitor RCTs Qualifying for ASCO-VF v2 Tail-of-the-Curve Bonus and ESMO-MCBS Immunotherapy-Triggered Long-term Plateau Adjustments in the Framework-Specified End Point by Cancer Type **eTable 5.** Kappa Correlation Coefficient of Reviewer Agreement Between RCTs That Displayed a Long-term Plateau According to the ESMO-MCBS v1.1 Criteria **eTable 6. **Included Studies
