# Supplementary material for: Comparison of Long-term Survival Benefits in Trials of Immune Checkpoint Inhibitor vs Non–Immune Checkpoint Inhibitor Anticancer Agents Using ASCO Value Framework and ESMO Magnitude of Clinical Benefit Scale
Source: JAMA Netw Open. 2019 Jul 10;2(7):e196803. doi: 10.1001/jamanetworkopen.2019.6803 (PMC6624800; doi:10.1001/jamanetworkopen.2019.6803)
Supplement: Supplement. — eFigure 1. ASCO-VF v2 Tail-of-the-Curve Bonus: PFS Percentage Improvement eFigure 2. ASCO-VF v2 Tail-of-the-Curve Bonus: OS Percentage Improvement eFigure 3. ESMO-MCBS v1.1 Immunotherapy-Triggered Long-term Plateau Adjustment: PFS Percentage Difference at Time Point Evaluated eFigure 4. ESMO-MCBS v1.1 Immunotherapy-Triggered Long-term Plateau Adjustment: OS Study Duration eTable 1. Primary Analysis of Risk Differences of Immune Checkpoint Inhibitor vs Non–Immune Checkpoint Inhibitor RCTs Qualifying for ASCO-VF v2 Tail-of-the-Curve Bonus and ESMO-MCBS Immunotherapy-Triggered Long-term Plateau Adjustments eTable 2. Sensitivity Analysis of Risk Differences of Immune Checkpoint Inhibitor vs Non–Immune Checkpoint Inhibitor RCTs Qualifying for ASCO-VF v2 Tail-of-the-Curve Bonus and ESMO-MCBS Immunotherapy-Triggered Long-term Plateau Adjustments eTable 3. Kappa Correlation Statistic for Agreement Between ASCO-VF v2 and ESMO-MCBS v1.1 Tail-of-the-Curve and Long-term Survival Bonuses eTable 4. Risk Differences of Immune Checkpoint Inhibitor vs Non–Immune Checkpoint Inhibitor RCTs Qualifying for ASCO-VF v2 Tail-of-the-Curve Bonus and ESMO-MCBS Immunotherapy-Triggered Long-term Plateau Adjustments in the Framework-Specified End Point by Cancer Type eTable 5. Kappa Correlation Coefficient of Reviewer Agreement Between RCTs That Displayed a Long-term Plateau According to the ESMO-MCBS v1.1 Criteria eTable 6. Included Studies [file jamanetwopen-2-e196803-s001.pdf]

Supplementary Online Content

Everest L, Shah M, Chan KKW. Comparison of long-term survival benefits in trials of immune checkpoint inhibitor vs non–immune checkpoint inhibitor anticancer agents using ASCO Value Framework and ESMO Magnitude of Clinical Benefit Scale. *JAMA Netw Open*. 2019;2(7):e196803. doi:10.1001/jamanetworkopen.2019.6803

eFigure 1. ASCO-VF v2 Tail-of-the-Curve Bonus: PFS Percentage Improvement

eFigure 2. ASCO-VF v2 Tail-of-the-Curve Bonus: OS Percentage Improvement

eFigure 3. ESMO-MCBS v1.1 Immunotherapy-Triggered Long-term Plateau Adjustment: PFS Percentage Difference at Time Point Evaluated

eFigure 4. ESMO-MCBS v1.1 Immunotherapy-Triggered Long-term Plateau Adjustment: OS Study Duration

eTable 1. Primary Analysis of Risk Differences of Immune Checkpoint Inhibitor vs Non–Immune Checkpoint Inhibitor RCTs Qualifying for ASCO-VF v2 Tail-of-the-Curve Bonus and ESMO-MCBS Immunotherapy-Triggered Long-term Plateau Adjustments

eTable 2. Sensitivity Analysis of Risk Differences of Immune Checkpoint Inhibitor vs Non–Immune Checkpoint Inhibitor RCTs Qualifying for ASCO-VF v2 Tail-of-the-Curve Bonus and ESMO-MCBS Immunotherapy-Triggered Long-term Plateau Adjustments

eTable 3. Kappa Correlation Statistic for Agreement Between ASCO-VF v2 and ESMO-MCBS v1.1 Tail-of-the-Curve and Long-term Survival Bonuses

eTable 4. Risk Differences of Immune Checkpoint Inhibitor vs Non–Immune Checkpoint Inhibitor RCTs Qualifying for ASCO-VF v2 Tail-of-the-Curve Bonus and ESMO-MCBS Immunotherapy-Triggered Long-term Plateau Adjustments in the Framework-Specified End Point by Cancer Type

eTable 5. Kappa Correlation Coefficient of Reviewer Agreement Between RCTs That Displayed a Long-term Plateau According to the ESMO-MCBS v1.1 Criteria

eTable 6. Included Studies

This supplementary material has been provided by the authors to give readers additional information about their work

© 2019 Everest L et al. *JAMA Network Open.*

eFigure 1. ASCO-VF v2 Tail-of-the-Curve Bonus: PFS Percentage Improvement

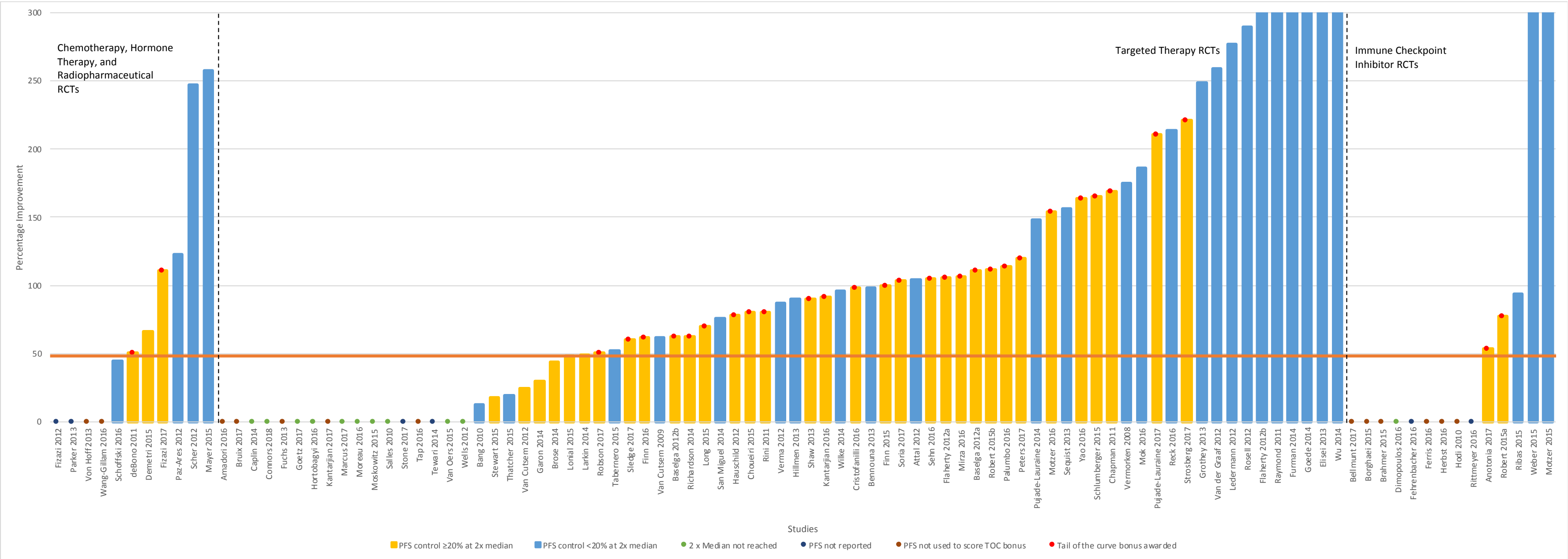

Blue bars indicate percentage improvement of the treatment arm over the control arm at time 2x the median, in RCTs that had less than 20% of patients surviving at time 2x median.

Yellow bars indicate percentage improvement of the treatment arm over the control arm at time 2x the median, in RCTs that had greater than or equal to 20% of patients surviving at time 2x median.

Green dots indicate RCTs that did not report PFS data at time 2x median.

Blue dots indicate RCTs that did not report a PFS KM curve.

Brown dots indicate RCTs that did not use PFS to evaluate the "tail of the curve" bonus.

Red dots indicate RCTs that were awarded with a "tail of the curve" bonus on the basis of PFS data.

© 2019 Everest L et al. *JAMA Network Open.*

eFigure 2. ASCO-VF v2 Tail-of-the-Curve Bonus: OS Percentage Improvement

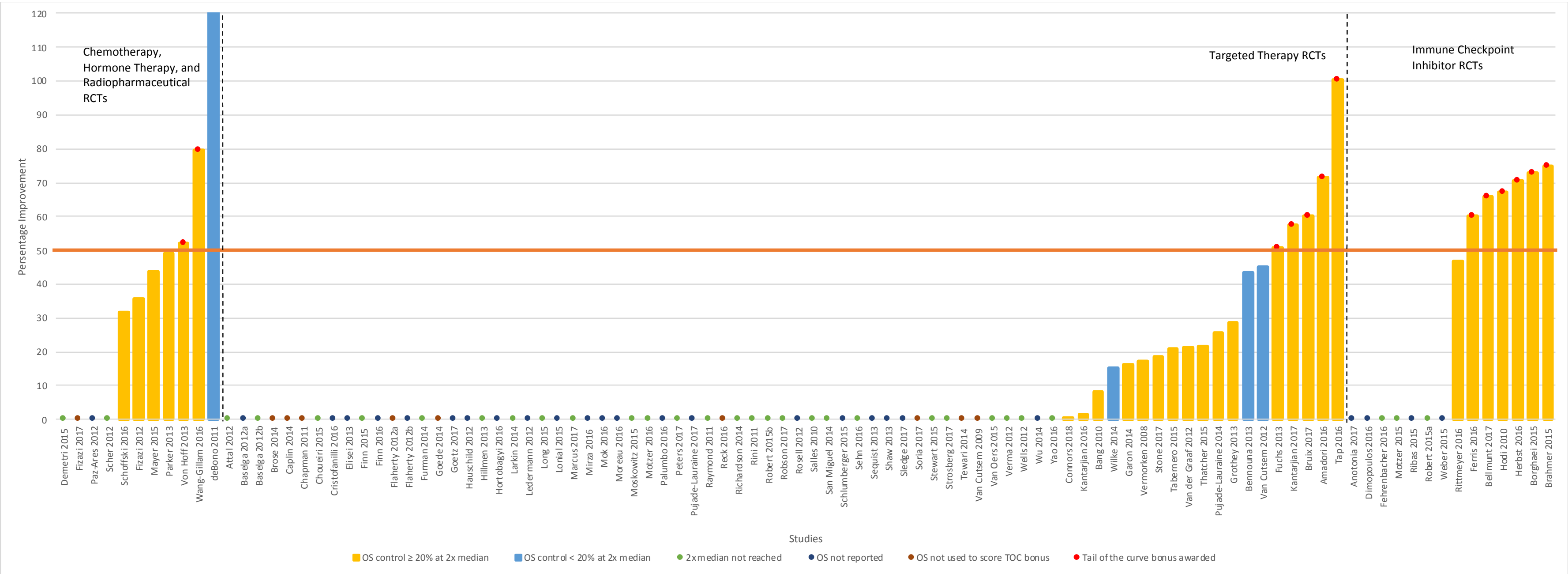

Blue bars indicate percentage improvement of the treatment arm over the control arm at time 2x the median, in studies that had less than 20% of patients surviving at time 2x median.
Yellow bars indicate percentage improvement of the treatment arm over the control arm at time 2x the median, in studies that had greater than or equal to 20% of patients surviving at time 2x median.
Green dots indicate RCTs that did not report OS data at time 2x median.
Blue dots indicate RCTs that did not report an OS KM curve.
Brown dots indicate RCTs that did not use OS to evaluate the “tail of the curve” bonus.
Red dots indicate RCTs that were awarded with a “tail of the curve” bonus on the basis of OS data.

© 2019 Everest L et al. *JAMA Network Open.*

eFigure 3. ESMO-MCBS v1.1 Immunotherapy-Triggered Long-term Plateau Adjustment: PFS Percentage Difference at Time Point Evaluated

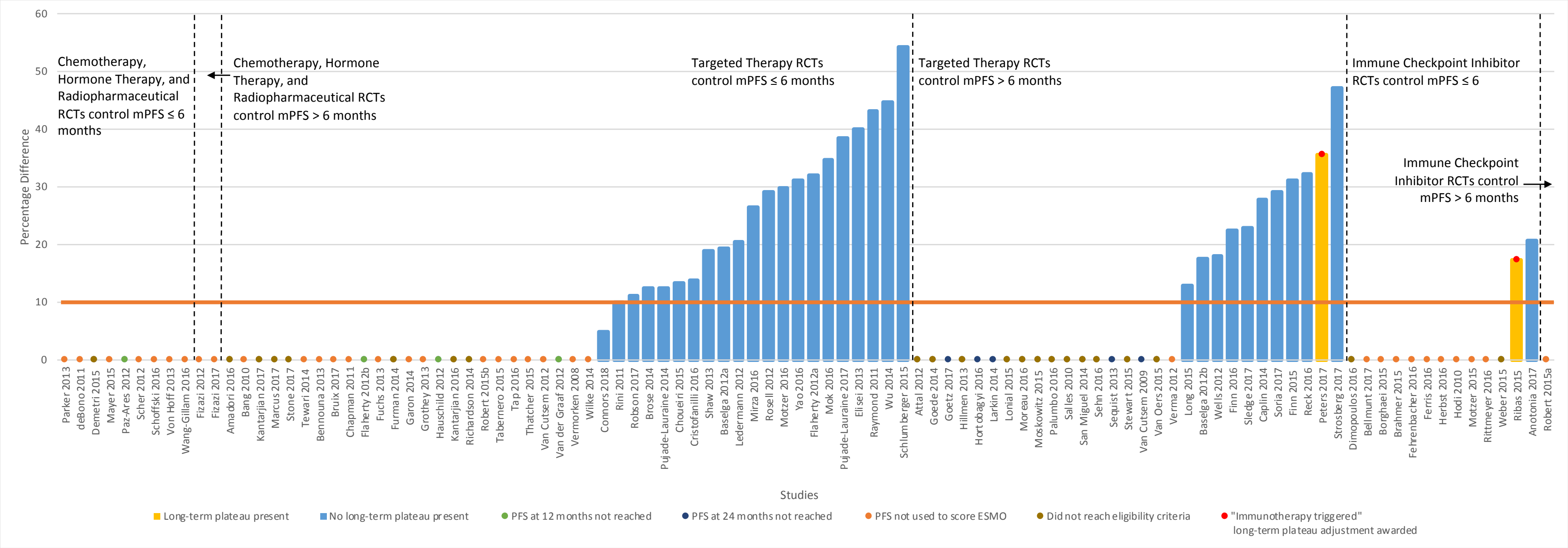

Blue bars indicate percentage difference in improvement of the treatment arm and control arm at 12 months or 24 months in RCTs that did not display a “long-term plateau”.
Yellow bars indicate percentage difference in improvement of the treatment arm and control arm at 12 months or 24 months in RCTs that did display a “long-term plateau”.
Green dots indicate RCTs that did not report PFS data at the time point assessed (12 months)
Dark blue dots indicate that the RCTs did not report PFS data at the time point assessed (24 months)
Orange dots indicate RCTs that did not use PFS data to assess the “immunotherapy triggered” long-term plateau adjustment
Brown dots indicate RCTs that did not meet eligibility criteria to be scored with the ESMO-MCBS.
Red dots indicate RCTs were awarded with the “immunotherapy triggered” long-term plateau adjustment based on PFS data.

© 2019 Everest L et al. *JAMA Network Open.*

eFigure 4. ESMO-MCBS v1.1 Immunotherapy-Triggered Long-term Plateau Adjustment: OS Study Duration

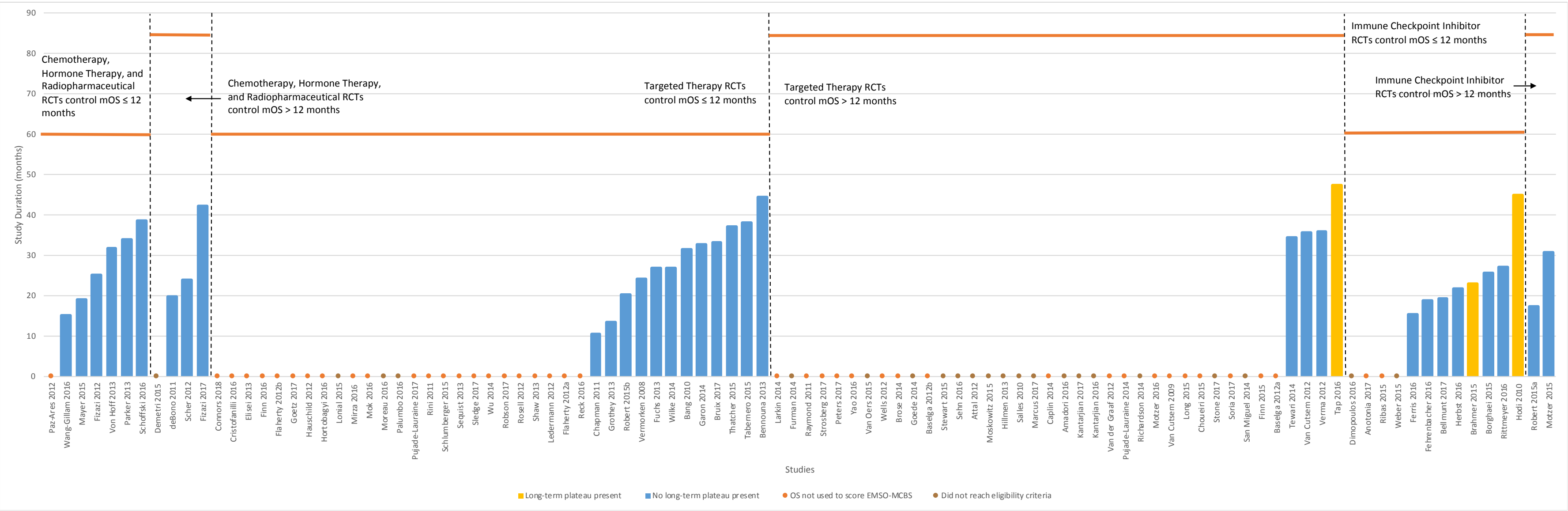

Blue bars indicate the study duration of RCTs that did not display a “long-term plateau”.
Yellow bars indicate the study duration of RCTs that did display a “long-term plateau”.
Orange dots indicate RCTs that did not use OS data to assess the “immunotherapy triggered” long-term plateau adjustment.
Brown dots indicate RCTs that did not meet eligibility criteria to be scored with the ESMO-MCBS.

© 2019 Everest L et al. *JAMA Network Open.*

eTable 1. Primary Analysis of Risk Differences of Immune Checkpoint Inhibitor vs Non–Immune Checkpoint Inhibitor RCTs Qualifying for ASCO-VF v2 Tail-of-the-Curve Bonus and ESMO-MCBS Immunotherapy-Triggered Long-term Plateau Adjustments

	**Endpoint**	**Framework**	**Therapy**	**Subgroup**	**Risk Difference**	**95% CI**	**p-value**
**Primary Analysis**	Framework-specified endpoint	ASCO-VF v2	Non-immune Checkpoint Inhibitor		0.14	-0.14, 0.42	0.32
				Chemotherapy, Hormone Therapy, and Radiopharmaceutical	0.12	-0.27,0.51	0.56
				Targeted Therapy	0.15	-0.14,0.43	0.32
		ESMO-MCBS v1.1	Non-immune Checkpoint Inhibitor		0.07	-0.09, 0.23	0.40
				Chemotherapy, Hormone Therapy, and Radiopharmaceutical	0.08	-0.07, 0.24	0.30
				Targeted Therapy	0.07	-0.09,0.23	0.42

ASCO-VF v2: American Society of Clinical Oncology Valuation Framework version 2.
ESMO-MCBS v1.1: European Society of Medical Oncology Magnitude of Clinical Benefit Scale version 1.1.
OS: Overall survival
PFS: Progression free survival
Framework-specified endpoint: the endpoint that ASCO-VF v2 and ESMO-MCBS v1.1 would use to assess respective long-term survival bonus/adjustments according to the framework algorithm, for an individual RCT.

© 2019 Everest L et al. *JAMA Network Open.*

eTable 2. Sensitivity Analysis of Risk Differences of Immune Checkpoint Inhibitor vs Non–Immune Checkpoint Inhibitor RCTs Qualifying for ASCO-VF v2 Tail-of-the-Curve Bonus and ESMO-MCBS Immunotherapy-Triggered Long-term Plateau Adjustments

	Endpoint	Framework	Therapy	Subgroup	Risk Difference	95% CI	p-value
**Sensitivity Analysis**	Overall survival	ASCO-VF v2	Non-immune Checkpoint Inhibitor		0.66	0.38, 0.94	<0.001
				Chemotherapy, Hormone Therapy, and Radiopharmaceutical	0.35	-0.07, 0.78	0.11
				Targeted Therapy	0.48	0.17, 0.80	0.003
		ESMO-MCBS v1.1	Non-immune Checkpoint Inhibitor		0	0, 0	NA
				Chemotherapy, Hormone Therapy, and Radiopharmaceutical	0	0, 0	NA
				Targeted Therapy	0	0, 0	NA
	Progression free survival	ASCO-VF v2	Non-immune Checkpoint Inhibitor		0.05	-0.10, 0.21	0.50
				Chemotherapy, Hormone Therapy, and Radiopharmaceutical	-0.04	-0.55, 0.46	0.87
				Targeted Therapy	-0.06	-0.46, 0.33	0.75
		ESMO-MCBS v1.1	Non-immune Checkpoint Inhibitor		0.48	-0.22, 1.17	0.18
				Chemotherapy, Hormone Therapy, and Radiopharmaceutical	0.5	-0.19, 1.19	0.16
				Targeted Therapy	0.48	-0.22, 1.17	0.18

ASCO-VF v2: American Society of Clinical Oncology Valuation Framework version 2.
ESMO-MCBS v1.1: European Society of Medical Oncology Magnitude of Clinical Benefit Scale version 1.1.
OS: Overall survival
PFS: Progression free survival

© 2019 Everest L et al. *JAMA Network Open.*

eTable 3. Kappa Correlation Statistic for Agreement Between ASCO-VF v2 and ESMO-MCBS v1.1 Tail-of-the-Curve and Long-term Survival Bonuses

Analysis	Endpoint Comparison	Cohen's Kappa Statistics			McNemar's chi-square test		Number of comparisons
		Kappa's Correlation Statistic*	95% CI	p-value	McNemar's chi-square statistic	p-value	
Primary Analysis	Framework-specified endpoint	0.01	-0.23, 0.22	0.50	32.24	<0.001	78
Sensitivity Analysis	Overall survival	0	-0.47, 0.47	0.50	9.09	0.003	35
	Progression free survival	0	-0.30, 0.30	0.50	16.41	<0.001	43
	Immune Checkpoint Inhibitor	-0.17	-0.56, 0.21	0.79	4.00	0.045	12
	Non-immune Checkpoint Inhibitor	0.04	-0.23, 0.30	0.39	27.03	<0.001	66

*Where 1 indicates perfect agreement and 0 indicates poor agreement.

© 2019 Everest L et al. *JAMA Network Open.*

eTable 4. Risk Differences of Immune Checkpoint Inhibitor vs Non–Immune Checkpoint Inhibitor RCTs Qualifying for ASCO-VF v2 Tail-of-the-Curve Bonus and ESMO-MCBS Immunotherapy-Triggered Long-term Plateau Adjustments in the Framework-Specified End Point by Cancer Type

Cancer Type	Framework	Risk Difference	95% CI	p-value	Number of RCTs	Number of ICI RCTs
Melanoma	ASCO-VF v2	-0.21	(-0.81, 0.37)	0.48	11	4
	ESMO-MCBS v1.1	0.25	(-0.17, 0.67)	0.22	11	4
Non-small cell lung cancer	ASCO-VF v2	0.27	(-0.19, 0.74)	0.25	17	7
	ESMO-MCBS v1.1	-0.1	(-0.28, 0.08)	0.29	17	7

RCTs: Randomized controlled trials
ICI: Immune checkpoint inhibitor

© 2019 Everest L et al. *JAMA Network Open.*

eTable 5. Kappa Correlation Coefficient of Reviewer Agreement Between RCTs That Displayed a Long-term Plateau According to the ESMO-MCBS v1.1 Criteria

	Kappa’s correlation coefficient	p-value	Comparisons
RCTs with PFS “long-term plateau”	0.718	<0.001	43
RCTs with OS “long-term plateau”	0.720	<0.001	35

Discrepancies between reviewers were found in 7 RCTs. Disagreements between reviewer assessment of the “long-term plateau” was resolved by consensus.
RCTs: Randomized controlled trials
OS: Overall survival
PFS: Progression free survival

© 2019 Everest L et al. *JAMA Network Open.*

eTable 6. Included Studies

First Author	FDA Notification Year	Indication	Therapy	Therapy Type	Primary Endpoint	HR OS	HR PFS	Median OS Difference (months)	Median PFS Difference (months)
Amadori	2016	Acute Myeloid Leukemia	Gemtuzumab	T	OS	0.69	NR	1.30	NR
Anotonia	2017	Non-Small Cell Lung Cancer	Durvalumab	I	OS/PFS	NR	0.52	NR	11.2
Attal	2012	Myeloma	Lenalidomide	T	PFS	1.25	0.50	NR	18.00
Bang	2010	Gastric Cancer	Trastuzumab	T + C	OS	0.74	0.71	2.70	1.20
Baselga	2012(a)	Advanced Breast Cancer	Everolimus	T	PFS	NR	0.36	NR	6.50
Baselga	2012(b)	Advanced Breast Cancer	Pertuzumab	T	PFS	0.64	0.62	NR	6.10
Bellmunt	2017	Advanced Urothelial Caner	Pembrolizumab	I	OS/PFS	0.71	0.98	2.90	-1.20
Bennouna	2013	Metastatic Colorectal Cancer	Bevacizumab	T	OS	0.81	0.67	1.40	1.6
Borghaei	2015	Non-Small Cell Lung Cancer	Nivolumab	I	OS	0.73	0.92	2.8	1.9
Brahmer	2015	Non-Small Cell Lung Cancer	Nivolumab	I	OS	0.59	0.62	3.2	0.7
Brose	2014	Metastatic Thyroid Cancer	Sorafenib	T	PFS	0.80	0.59	NR	5.00
Bruix	2017	Hepatocellular Carcinoma	Regorafenib	T	OS	0.63	0.46	2.80	1.60
Caplin	2014	Entero-pancreatic Endocrine Tumours	Lanreotide	T	PFS	NR	0.47	NR	9.00
Chapman	2012	Metastatic Melanoma	Vemurafenib	T	OS/PFS	0.37	0.26	2.20	3.70
Chouieri	2015	Metastatic Renal Cell Carcinoma	Cabozantinib	T	PFS	0.67	0.58	-2.80	3.60
Connors	2018	Hodgkin’s Lymphoma	Brentuximab Vedotin	T + C	PFS	0.73	0.77	NR	NR
Cristofanill	2016	Metastatic Breast Cancer	Palbociclib	T	PFS	NR	0.46	NR	4.90
De Bono	2010	Metastatic Castration Resistant Prostate Cancer	Cabazitaxel	C	OS	0.70	0.74	2.40	1.40
Demetri	2016	Leiomyosarcoma	Trabectedin	C	OS	0.87	0.55	0.50	2.70
Dimopoulos	2016	Multiple Myeloma	Daratumumab	I	PFS	NR	0.37	NR	2.50
Elisei	2013	Medullary Thyroid Cancer	Cabozantinib	T	PFS	NR	0.28	NA	7.20
Fehrenbacher	2016	Non-Small Cell Lung Cancer	Atezolizumab	I	OS	0.73	0.94	2.9	0.3
Ferris	2016	Metastatic Head and Neck Carcinoma	Nivolumab	I	OS	0.70	0.89	2.40	0.30
Finn	2015	Advanced Breast Cancer	Palbocicib	T	PFS	NR	0.49	NR	10
Finn	2016	Metastatic Breast Cancer	Palbociclib	T	PFS	NR	0.58	NR	10.30
Fizazi	2012	Metastatic Castration Resistant Prostate Cancer	Abiraterone	H	OS	0.74	0.66	4.60	1.90
Flaherty	2012(a)	Metastatic Melanoma	Trametinib	T	PFS	0.54	0.45	NR	3.30
Flaherty	2012(b)	Metastatic Melanoma	Trametinib	T	PFS	NR	0.56	NR	3.4
Fuchs	2013	Advanced Gastric Cancer	Ramucirumab	T	OS	0.78	0.48	1.40	0.80
Furman	2014	Chronic Lymphocytic Leukemia	Idelalisib	T	PFS	0.28	0.15	NA	8.5
Garon	2014	Non-Small Cell Lung Cancer	Ramucirumab	T	OS	0.86	0.76	1.40	1.50
Goede	2014	Chronic Lymphocytic Leukemia	Obinutuzumab	T	PFS	0.41	0.18	NA	15.60
Goetz	2017	Advanced Breast Cancer	Abemaciclib	T	PFS	NR	0.51	NR	NR
Grothey	2013	Metastatic Colorectal Cancer	Regorafenib	T	OS	0.77	0.49	1.40	0.20
Hauschild	2012	Metastatic Melanoma	Dabrafenib	T	PFS	0.61	0.30	NR	2.40

© 2019 Everest L et al. *JAMA Network Open.*

Herbst	2016	Non-Small Cell Lung Cancer	Pembrolizumab	T	PFS	0.61	0.79	1.90	0.10
Hillmen	2013	Chronic Lymphocytic Leukemia	Ofatumumab	T	PFS	0.91	0.57	NR	9.30
Hodi	2010	Metastatic Melanoma	Ipilimumab	I	OS	0.68	0.81	3.60	0.00
Hortobagyi	2012	Advanced Breast Cancer	Ribociclib	T	PFS	NR	0.56	NR	7.30
Kantarjian	2016	Acute Lymphoblastic Leukemia	Inotuzumab	T	OS	0.77	0.45	0.60	3.20
Kantarjian	2017	Acute Lymphoblastic Leukemia	Blinatumomab	T	OS	0.71	NR	3.70	NR
	2014	Metastatic Melanoma	Cobimetinib	T	OS/PFS	0.65	0.51	NR	3.7
Ledermann	2012	Ovarian Cancer	Olaparib	T	PFS	0.94	0.35	NR	3.6
Long	2015	Melanoma	Trametinib	T	PFS	0.71	0.67	6.4	2.2
Lonial	2015	Multiple Myeloma	Elotuzumab	T	PFS	NR	0.70	NA	4.5
Marcus	2017	Non-Hodgkin's Lymphoma	Obinutuzumab	T	PFS	0.75	0.66	NR	NR
Mayer	2015	Metastatic Colorectal Cancer	TAS-102	C	OS	0.68	0.48	1.80	0.30
Miller	2007	Metastatic Breast Cancer	Bevacizumab	T	PFS	0.88	0.60	1.50	5.90
Mirza	2016	Ovarian Cancer	Niraparib	T	PFS	NR	0.27	NR	5.60
Mok	2016	Non-Small Cell Lung Cancer	Osimertinib	T	PFS	NR	0.30	NR	5.70
Moreau	2016	Multiple Myeloma	Ixazomib	T	PFS	NR	0.74	NR	5.90
Moskowitz	2015	Hodgkin’s Lymphoma	Brentuximab Vedotin	T	PFS	1.15	0.57	NR	18.80
Motzer	2015	Metastatic Renal Cell Carcinoma	Nivolumab	I	OS	0.73	0.88	5.40	0.20
Motzer	2016	Metastatic Renal Cell Carcinoma	Lenvatinib + Everolimus	T	PFS	0.51	0.40	9.0	8.1
Palumbo	2016	Multiple Myeloma	Daratumumab	T	PFS	NR	0.39	NR	7.80
Parker	2013	Metastatic Prostate Cancer	Alpha Emitter Radium-223	R	OS	0.70	NR	3.6	NR
Paz-Ares	2013	Non-Small Cell Lung Cancer	Pemetrexed	C	PFS	0.78	0.60	2.90	1.30
Peters	2017	Non-Small Cell Lung Cancer	Alectinib	T	PFS	0.76	0.47	NR	15.30
Pujade-Lauraine	2014	Ovarian Cancer	Avastin	T	PFS	0.85	0.48	3.30	3.30
Pujade-Lauraine	2017	Ovarian Cancer	Olaparib	T	PFS	NR	0.30	NR	13.60
Raymond	2011	Pancreatic Islet Cell Tumors	Sunitinib	T	PFS	0.41	0.42	NR	5.90
Reck	2016	Non-Small Cell Lung Cancer	Nintedanib	T	PFS	0.94	0.79	1.00	1.30
RIbas	2015	Ipilimumab-refractory Melanoma	Pembrolizumab	T	PFS	NR	0.57	NR	0.20
Richardson	2014	Multiple Myeloma	Pomalidomide + Dexamethasone	T	PFS	0.94	0.68	2.9	1.6
Rini	2011	Metastatic Renal Cell Carcinoma	Axitinib	T	PFS	NR	0.67	NR	2.00
Rittmeyer	2016	Non-Small Cell Lung Cancer	Atezolizumab	I	OS	0.73	0.95	4.20	-1.20
Robert	2015(a)	Advanced Melanoma	Dabrafenib	T	OS	0.69	0.56	3.80	4.10
Robert	2015(b)	Advanced Melanoma	Pembrolizumab	I	OS/PFS	0.69	0.58	NR	1.30
Rosell	2012	Non-Small Cell Lung Cancer	Tarceva	T	PFS	1.04	0.37	-0.20	4.20
Salles	2012	Follicular Lymphoma	Rituximab	T	PFS	0.87	0.55	NR	5.70
San-Miguel	2014	Multiple Myeloma	Panobinostat	T	PFS	0.87	0.63	3.25	3.91
Scher	2012	Metastatic Castration Resistant Prostate Cancer	Enzalutamide	H	OS	0.63	0.40	13.60	2.90
Schlumberger	2015	Thyroid Cancer	Lenvatinib	T	PFS	0.62	0.21	NR	14.70
Schoffski	2016	Soft Tissue Sarcoma	Eribulin	C	OS	0.77	0.88	2.00	0.00

© 2019 Everest L et al. *JAMA Network Open.*

Sehn	2016	Non-Hodgkin's Lymphoma	Obinutuzumab	T	PFS	0.82	0.52	NR	33.10
Sequist	2013	Non-Small Cell Lung Cancer	Afatinib	T	PFS	NR	0.58	NR	4.64
Shaw	2013	Non-Small Cell Lung Cancer	Crizotinib	T	PFS	1.02	0.49	-2.50	4.70
Sledge	2017	Advanced Breast Cancer	Abemaciclib	T	PFS	NR	0.55	NR	10.20
Soria	2017	Non-small Cell Lung Cancer	Ceritinib	T	PFS	0.73	0.55	7.80	8.50
Stewart	2014	Multiple Myeloma	Carfilzomib	T	PFS	0.79	0.69	0.00	8.70
Stone	2017	Acute Myeloid Leukemia	Midostaurin	T	OS	0.78	NR	49.10	NR
Strosberg	2017	Midgut Neuroendocrine Tumors	Lutetium-177- Dotatate	T	PFS	0.40	0.21	NR	16.6
Tabernero	2015	Metastatic Colorectal Cancer	Ramucirumab	T	OS	0.84	0.79	1.60	1.20
Tewari	2014	Advanced Cervical Cancer	Bevacizumab	T	OS	0.68	NR	3.20	NR
Thatcher	2015	Non-Small Cell Lung Cancer	Necitumumab	T	OS	0.84	0.85	1.60	0.20
Van Cutsem	2009	Metastatic Colorectal Cancer	Cetuximab	T	PFS	0.93	0.85	1.30	0.90
Van Cutsem	2012	Metastatic Colorectal Cancer	Aflibercept	T	OS	0.82	0.76	0.90	2.23
Van der Graaf	2012	Soft Tissue Sarcoma	Pazopanib	T	PFS	0.86	0.31	1.80	3.00
Van Oers	2015	Chronic Lymphocytic Leukemia	Ofatumumab	T	PFS	0.85	0.50	NR	14.20
Verma	2012	Metastatic Breast Cancer	Trastuzumab	T + C	OS/PFS	0.68	0.65	5.80	3.20
Vermorken	2008	Head and Neck Cancer	Cetuximab	T	OS	0.80	0.54	2.70	2.30
Von Hoff	2013	Metastatic Pancreatic Cancer	Paclitaxel	C	OS	0.72	0.69	1.80	1.80
Wang-Gillam	2016	Metastatic Pancreatic Cancer	Irinotecan	C	OS	0.67	0.56	1.90	1.60
Weber	2015	Advanced Melanoma	Nivolumab	I	OS/PFS	NR	0.82	NR	0.43
Wells	2012	Metastatic Medullary Thyroid Cancer	Vandetanib	T	PFS	0.89	0.46	NR	10.70
Wilke	2014	Gastric Adenocarcinoma	Ramucirumab	T	OS	0.81	0.64	2.20	1.50
Wu	2014	Advanced Non-Small-Cell Lung Cancer	Afatinib	T	PFS	NR	0.28	NR	5.4
Yao	2016	Neuroendocrine Tumors	Everolimus	T	PFS	0.64	0.48	NR	8.50

T, Targeted Agent; C, Chemotherapy; I, Immunotherapy; H, Hormone therapy; R, Radiopharmaceutical; OS, Overall survival; PFS, Progression-free survival; TTP, Time to progression; NR, Not Reported.

© 2019 Everest L et al. *JAMA Network Open.*
